# Virtual Reality for Shoulder Rehabilitation: Accuracy Evaluation of Oculus Quest 2

**DOI:** 10.3390/s22155511

**Published:** 2022-07-23

**Authors:** Arianna Carnevale, Ilaria Mannocchi, Mohamed Saifeddine Hadj Sassi, Marco Carli, Giovanna De Luca, Umile Giuseppe Longo, Vincenzo Denaro, Emiliano Schena

**Affiliations:** 1 Research Unit of Orthopaedic and Trauma Surgery, Fondazione Policlinico Universitario Campus Bio-Medico, Via Alvaro del Portillo, 200, 00128 Roma, Italy; arianna.carnevale@unicampus.it (A.C.); giovanna.deluca1398@gmail.com (G.D.L.); denaro@unicampus.it (V.D.); 2 Research Unit of Orthopaedic and Trauma Surgery, Department of Medicine and Surgery, Università Campus Bio-Medico di Roma, Via Alvaro del Portillo, 21, 00128 Roma, Italy; 3 Laboratory of Measurement and Biomedical Instrumentation, Department of Engineering, Università Campus Bio-Medico di Roma, Via Alvaro del Portillo, 21, 00128 Rome, Italy; e.schena@unicampus.it; 4 Department of Industrial, Electronic and Mechanical Engineering, University of Roma Tre, Via Vito Volterra, 62, 00146 Rome, Italy; ilaria.mannocchi@uniroma3.it (I.M.); mohamedsaifeddine.hadjsassi@uniroma3.it (M.S.H.S.); marco.carli@uniroma3.it (M.C.)

**Keywords:** virtual reality, Oculus Quest 2, upper limb, rehabilitation, shoulder

## Abstract

Virtual reality (VR) systems are becoming increasingly attractive as joint kinematics monitoring systems during rehabilitation. This study aimed to evaluate the accuracy of the Oculus Quest 2 in measuring translational and rotational displacements. As the Oculus Quest 2 was chosen for future applications in shoulder rehabilitation, the translation range (minimum: ~200 mm, maximum: ~700 mm) corresponded to the forearm length of the 5th percentile female and the upper limb length of the 95th percentile male. The controller was moved on two structures designed to allow different translational displacements and rotations in the range 0–180°, to cover the range of motion of the upper limb. The controller measures were compared with those of a Qualisys optical capture system. The results showed a mean absolute error of 13.52 ± 6.57 mm at a distance of 500 mm from the head-mounted display along the x-direction. The maximum mean absolute error for rotational displacements was found to be 1.11 ± 0.37° for a rotation of 40° around the z-axis. Oculus Quest 2 is a promising VR tool for monitoring shoulder kinematics during rehabilitation. The inside-out movement tracking makes Oculus Quest 2 a viable alternative to traditional motion analysis systems.

## 1. Introduction

Recent developments in Virtual Reality (VR) have been applied in medical and rehabilitation areas and different devices have been used to detect and monitor human movement [1,2]. Gaming technologies, such as Microsoft Kinect and Nintendo Wii, have been included in the patient’s rehabilitation protocol, having positive results [3,4,5,6]. Furthermore, there has been a rapid increase in the use of VR systems as they have become more affordable [7]. In VR, the user is fully immersed in a virtual space and can interact with virtual objects, in simulated environments, using controllers [7]. These technologies can also track human movements and could engage the patient to increase their adherence to the rehabilitation protocol [8]. The immersivity of these devices allows to experience different virtual contexts, fostering an interactive patient experience. This could make long-term rehabilitation more enjoyable and fun [7]. In fact, VR has been used for the recovery of different clinical conditions [9,10,11,12]. In the orthopedic field, rehabilitation has a crucial importance after surgery or traumatic events for the recovery of the compromised functionalities [8]. Among shoulder musculoskeletal disorders, rotator cuff tears have a high incidence [13,14]. In [15], a new approach for the right shoulder’s rotator cuff is presented. The authors used Oculus Rift DK2 and a motion tracking system (Intel Real Sense) to design a game considering the movements of elevation and abduction. The system was evaluated by four experts, who concluded that the application could be used for upper limb rehabilitation. 

Currently, the assessment of patients’ shoulder clinical condition is typically done through qualitative methodologies by filling out questionnaires and clinical scales related to the specific pathology [16,17,18]. Meanwhile, these new devices allow quantitative evaluation of the movements performed by the patient, thus enabling real-time feedback on action correction. However, to be effective, it is mandatory to evaluate the tracking system before its application in a rehabilitation trial [19]. Usually, clinical and digital goniometers are the most used devices for measuring shoulder joints ROM [20]. Nevertheless, the Microsoft Kinect system could be a good alternative. In [20,21], the measures of shoulder functional movements, provided by Kinect, have been compared with the measure given by clinical and digital goniometers and optoelectronic system with good results in terms of device reliability and validity. In a recent systematic review [22], the authors evidenced moderate to good levels of intra- and inter-rater reliability to measure shoulder’s ROM using different devices, such as smartphone applications, digital inclinometers/goniometers, inertial sensors, and Kinect.

Other VR devices, such as Oculus Rift S and Oculus Touch, have been evaluated for rehabilitation purposes, comparing them to an optoelectronic system [23,24]. The results highlighted a notable accuracy and precision of these devices during different translations and rotations. The performance evaluation of a VR motion tracking device is crucial to evaluate their appropriateness for research and clinical applications. The performance evaluation of a pose tracking device can be performed by assessing particular tracking device’s quality metrics, such as translational and rotational accuracy. The controllers of Oculus Quest 2 are peripheral accessories serving as end-effectors of the upper limb kinematic chain. The pose (i.e., position and orientation) of the end-effector in the 3D space can be entirely described by using six degrees of freedom (DoF), i.e., three DoF for translations and three DoF for rotations.

The goal of this work was to evaluate, for the first time, the accuracy of the VR system Oculus Quest 2 in measuring translational and rotational displacements. To this aim, a custom app has been developed by using Unity and the Oculus Integration Package. An optoelectronic system has also acquired the translational and rotational displacements to compare measurements with those of Oculus Quest 2.

The rest of the article is organized as follows: Section 2 presents the methodological approach used, i.e., the experimental design and data analysis, and Section 3 presents the results, which are then discussed in Section 4.

## 2. Materials and Methods

### 2.1. Experimental Setup

**Figure 1:**
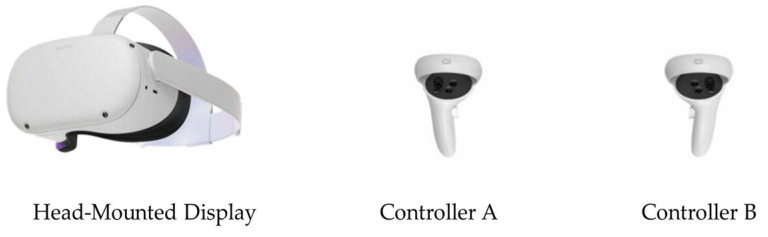
Oculus Quest 2. From the left, the head-mounted display, the left controller (Controller A), and right controller (Controller B).

An Oculus Quest 2 Head-Mounted Display (HMD) and two Oculus Touch Controllers (Figure 1) were employed in this study. In Table 1, the main technical features of the Oculus Quest 2 VR system are reported. A custom application was implemented in Unity (Unity, v. 2020.17) to register and store the controller’s position and orientation at a sampling rate of 200 Hz.

A Qualisys™ Optical Motion Capture (OMC) system (Qualisys AB, Gothenburg, Sweden) equipped with two Miqus Video (sampling rate, 25 Hz) and ten Miqus M3 cameras (sampling rate, 100 Hz) was used as a reference system. The manufacturer reported for Miqus M3 cameras a 3D resolution of 0.11 mm and a maximum capture distance equal to 15 m [25].

**Figure 2:**
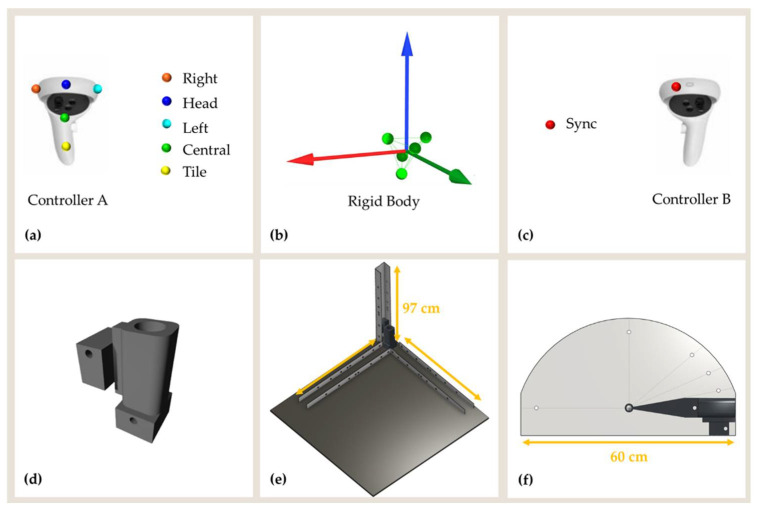
(**a**) Controller A and markers configuration used to define the (**b**) rigid body. (**c**) Controller B and position of the synchronization marker. Controller A was allocated in a (**d**) custom-made support designed to be easily moved on the structures for (**e**) translational and (**f**) rotational movements.

The two Miqus Video cameras were used to have synchronized videos available for use in subsequent analyses as a reference. The OMC was employed to capture the pose (position and orientation) of the geometric center of the rigid body defined in Qualisys Track Manager (QTM) software (v. 2021.2, Build 6720). In particular, five photo-reflective markers (diameter, 8 mm) were positioned on the controller A as shown in Figure 2a,b. A single marker was positioned on controller B (Figure 2c). As explained later, this marker was used for the synchronization of Oculus Quest 2 and OMC signals. A custom 3D support was printed in polylactic acid (PLA) filaments by using the 3D printer Ultimaker S2+ (Figure 2d). The support has been designed and appropriately sized to host the controller A during the experimental tests. Figure 2e,f show two structures designed for allowing translational and rotational movements.

### 2.2. Experimental Procedure

All measurements were performed by securing the left-hand controller (controller A) in the handmade holder, while a Healthy Volunteer (HV) held controller B during the experiments. Before each trial, the HV performed an initial calibration to set the 3D virtual space, i.e., the play area where they can safely move. Then, HV was asked to perform quick movements with the controller B (i.e., the one with the synchronization marker on it) at the beginning and end of each trial for signal synchronization.

#### 2.2.1. Translational Movements

Measurements were run along each 3D axis (x–width, y–depth, and z-height). A custom structure was used to move controller A along each axis (Figure 2e). As the Oculus Quest 2 was chosen for future applications in shoulder rehabilitation, the minimum (approximately 200 mm) and maximum (approximately 700 mm) distances analyzed were those corresponding to the forearm length of the 5th percentile female and the upper limb length of the 95th percentile male, respectively, and thus, hand length was not considered [26,27].

**Figure 3:**
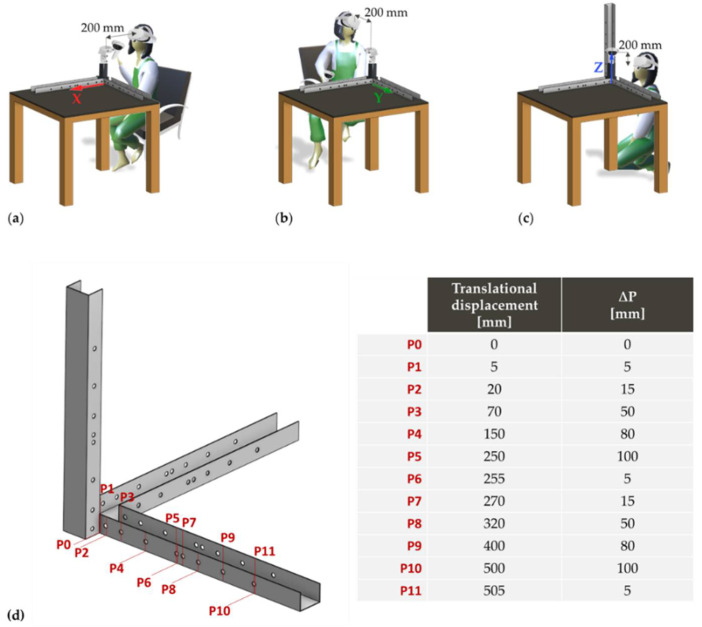
Acquisition configuration of translational displacements along the (**a**) x-axis, (**b**) y-axis, and (**c**) z-axis, starting at a distance of about 200 mm from the reference position P0; (**d**) representation of translational displacements P_i_ (i = 1, …,11) and variations (ΔP, i.e., differences between translational displacements $i^{\mathrm{th}}$ and $i^{\mathrm{th}-1}$.

All the translational displacements started with the HMD at 200 mm from the origin (P_0_) of the reference system until the maximum distance (P_11_ = 705 mm) at variable steps, as shown in Figure 3. At P_0_, controller A was left stationary for about 20 s and at subsequent positions P_i_ (i = 1,…,11) for about 10 s. At the positions P_1_ (5 mm), P_6_ (255 mm), and P_11_ (505 mm), an increment of 5 mm related to the previous positions (P_0_—0 mm, P_5_—250 mm, and P_10_—500 mm, respectively) was evaluated, to appreciate the Oculus Quest 2’s capability to detect small variations at distances gradually increasing than the position of the HMD. Acquisitions were repeated seven times along each axis, for a total of N = 252 (12 positions × 7 repetitions × 3 axis) static samples.

#### 2.2.2. Rotational Movements

**Figure 4:**
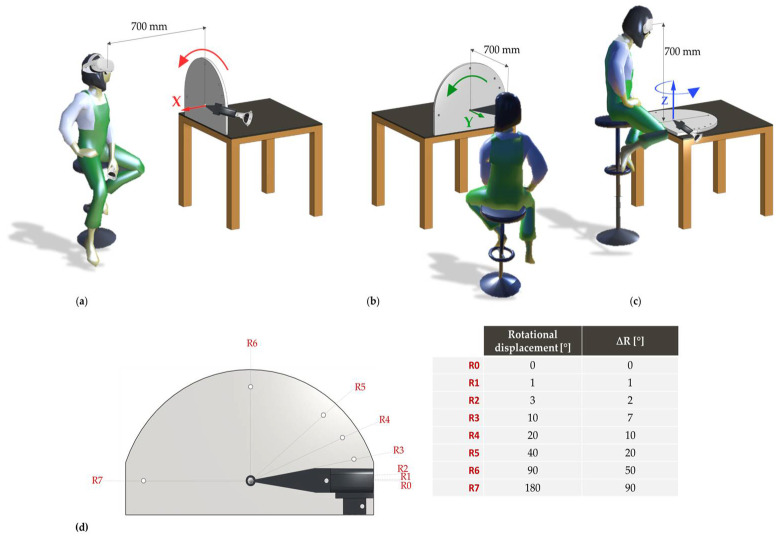
Acquisition configuration of rotational displacements around the (**a**) x-axis, (**b**) y-axis, and (**c**) z-axis, starting at a distance of about 700 mm from the reference position R0; (**d**) representation of rotational displacements R_i_ (i = 1,…,11) and variations (ΔR, i.e., differences between rotational displacements $i^{\mathrm{th}}$ and $i^{\mathrm{th}-1}$.

A custom structure was realized to move controller A around each axis (x—roll, y—pitch, and z—yaw) (Figure 4). The selected rotation interval ranged from 0° to 180° to cover the range of motion of the upper limb [28]. All the rotational displacements were performed by placing the HMD at a distance of about 700 mm from controller A during all the rotational displacements in the range 0°–180°. A total of eight rotational displacements ($R_{i}$, with i = 0,…,7) were reached by controller A seven times in every task, returning at each repetition to the starting position $R_{0}$, for a total of 14 rotational displacements. At each $R_{i}$, controller A was left stationary for 5 s. A total of N = 294 (14 rotational displacements × 7 repetitions × 3 axes) static samples were acquired.

### 2.3. Data Analysis

Preprocessing (labeling and gap-filling) of markers trajectories was performed in QTM. A careful analysis of the recorded videos allowed the recognition of the start and end events of the *i*-th positions. As mentioned above, tracking the pose of controller A with the OMC was performed by defining a rigid body in QTM, and in this way, the 6 degrees of freedom tracking were evaluated (i.e., translations and rotations along and around each 3D axes, respectively).

Data analyzed in QTM were processed using MATLAB. As sampling rates of data acquisition systems were different (Qualisys—100 Hz, Oculus Quest 2–200 Hz), resampling at the minimum sampling rate was performed. As Unity uses a left-handed coordinate system, while Qualisys uses a right-handed coordinate system, data from controllers were expressed with respect to the OMC coordinate system. Moreover, the orientation of the controller A was reported as a quaternion in Unity, so a conversion from quaternion to rotation matrix was performed.

A common event of quick upper limb elevation was used to synchronize the system under test (i.e., Oculus Quest 2) and the reference one (i.e., Qualisys™). In particular, the synchronization events were identified in correspondence with the maximum values of the trajectories of the Sync marker at the beginning and the end of all the trials. Maximum values corresponded to the maximum positions reached with controller B by the HV during the quick elevation movement of the upper limb.

After signals synchronization, both translations and rotations signals were windowed to select 3 s between two consecutive events. The selected windows of each position and rotation were defined assuming a steady-state condition reached by controller A without artifacts related to the movements required to move the controller.

#### 2.3.1. Translational Accuracy

Data were averaged on the 3 s of recording to compute the mean measured positions at each translational displacement performed. The absolute positional error $e_{P}$ was calculated as follows:

(1) $$e_{P}=\left|p^{Q\;}-p^{C}\right|\;$$

where $p^{Q}$ is the position measured by the Qualisys OMC system and $p^{C\;}$ is the position measured by the controller.

The positional percentage error $e_{P,\%}$ was evaluated as follows:

(2) $$e_{P,\%}=\frac{e_{p}}{p^{Q}}\cdot 100\;\;$$

The error $e^{\Delta }$ corresponding to the smaller step size (i.e., 5 mm) performed at P0 (0 mm), P5 (250 mm), and P10 (500 mm) was computed as follows:

(3) $$e^{\Delta }=\Delta Q-\Delta C\;$$

where ΔQ and ΔC were the steps measured by the Qualisys OMC system and the Oculus Quest 2, respectively, between the position P_i+1_ and P_i_.

#### 2.3.2. Rotational Accuracy

The mean rotation matrix was computed at each rotational displacement reached by both Oculus Quest 2 and the OMC systems. The rotation matrix M relating the starting ($\;R_{0,i}$) and ending ($R_{i}^{T}$) rotation matrices was computed as follows [23]:

(4) $$M=R_{0,i}R_{i}^{T},\;i=1,\ldots ,7$$

The final rotation angle Θ was computed as follows:

(5) trM=1+2cosΘ

then,

(6) $$\Theta =arccos\left(\frac{trM-1}{2}\right)$$

The absolute rotational error $e_{R}$ was calculated as follows:

(7) $$e_{R}=\left|\Theta^{C\;}-\Theta^{Q}\right|\;$$

where $\Theta^{Q}$ is the rotation measured by the Qualisys OMC system and $\Theta^{C\;}$ is the rotation angle measured by controller A.

The rotational percentage error $e_{R,\%}$ was evaluated as follows:

(8) $$e_{R,\%}=\frac{e_{R}}{\Theta^{Q}}\cdot 100\;\;$$

For both translational and rotational displacements, Bland–Altman analysis was performed to quantify the degree of agreement between the two measurement systems by defining the 95% limits of agreement (LOA), which estimates the interval where 95% of the differences between both systems fall [29]. LOA were expressed as MOD ± 1.96⋅SD where MOD represents the mean of difference and SD the standard deviation of the difference between the Qualisys OMC and Oculus Quest 2.

## 3. Results

### 3.1. Translational Accuracy

**Figure 5:**
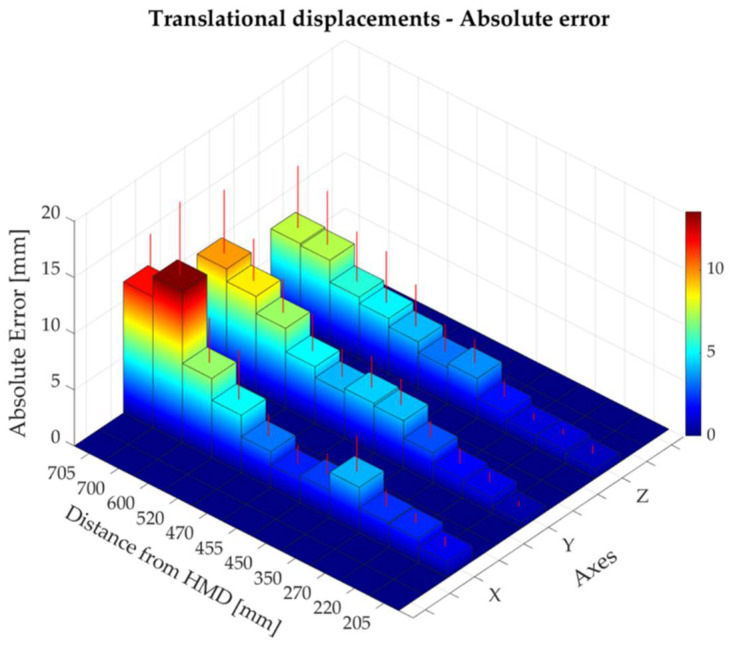
Absolute error along the x-, y-, and z-axes at different distances from the head-mounted display (HMD).

**Figure 6:**
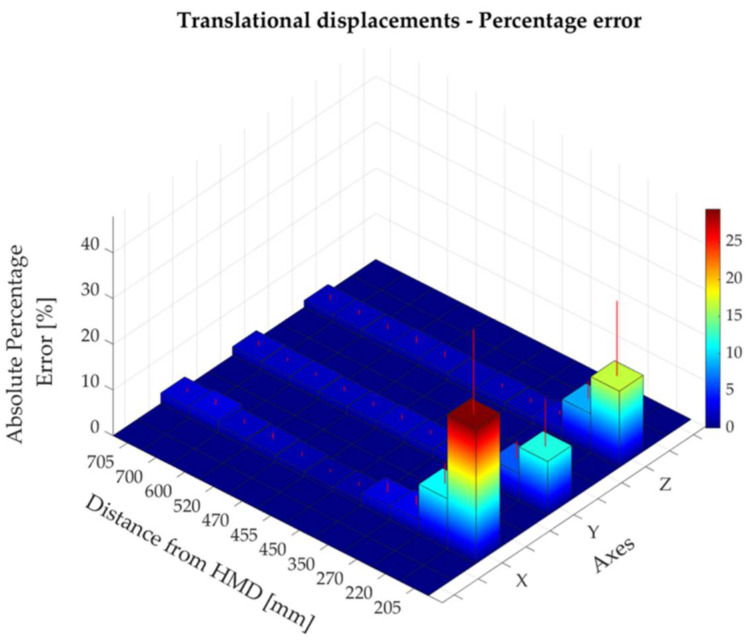
The percentage error along the x-, y-, and z-axes at different distances from the head-mounted display (HMD).

**Figure 7:**
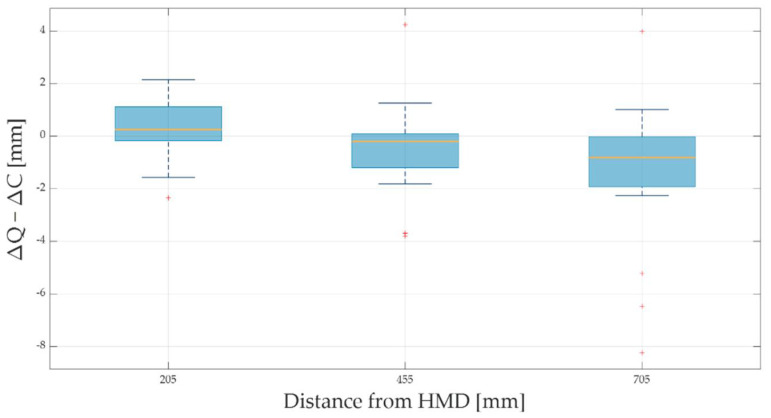
Error corresponding to the increment of 5 mm at the distances of 205 mm, 455 mm, and 705 mm from the head-mounted display (HMD).

**Figure 8:**
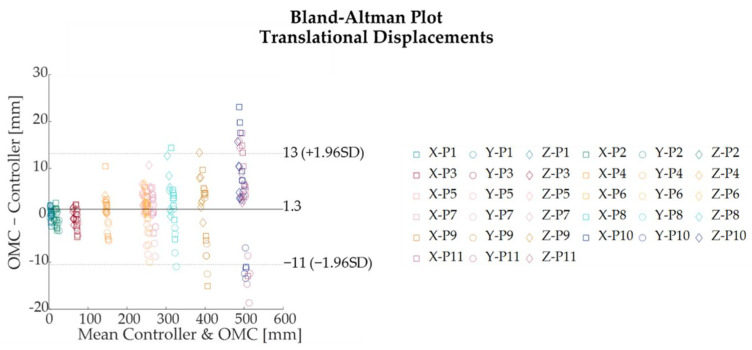
Bland–Altman plots comparing the translational displacements measured by the Qualisys Optical Motion Capture (OMC) system and by the Controller A of the Oculus Quest 2.

The absolute and percentage errors corresponding to all the translational displacements performed along the x-, y-, and z- directions are reported in Table 2. The maximum absolute error—expressed as mean ± standard deviation—was found to be 13.52 ± 6.57 mm along the x-axis at a distance of about 700 mm from the HMD, corresponding to a translational displacement of 500 mm from the starting position of controller A (i.e., at about 200 mm from the HMD). Along x-, y-, and z-directions, the absolute errors increased as the distance of the controller A from the HMD raised (Figure 5). The highest percentage errors were found to be 29.5%, 11.8%, and 16.7% for the x-, y-, and z-axes, respectively (Table 2, Figure 6). These results corresponded to the smallest increments (i.e., 5 mm) from the starting position, at which an absolute error of 1.38 mm, 0.55 mm, and 0.81 mm was found for the x-, y-, and z-axes, respectively. The results showed that the Oculus Quest 2 was able to discriminate the smallest step size of 5 mm at the distances of 205 mm, 455 mm, and 705 mm from the HMD, although the performance was better at shorter distances (Figure 7). Bland–Altman analysis for the translational displacements confirmed that the performances of Oculus Quest 2 decreased at higher distances, as shown in Figure 8 by the increasing dispersion of the differences between the two measurement systems as the distance of controller A from the HMD increases.

### 3.2. Rotational Accuracy

**Figure 9:**
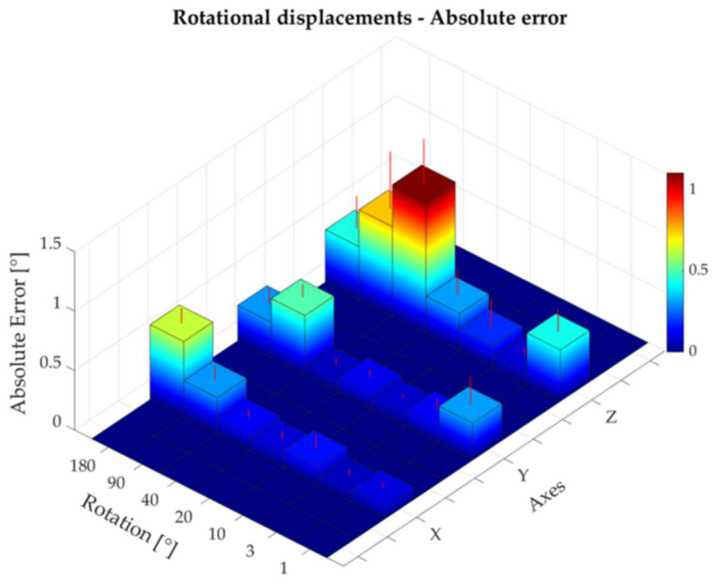
Absolute error corresponding to rotational displacements around the x-, y-, and z-axes.

**Figure 10:**
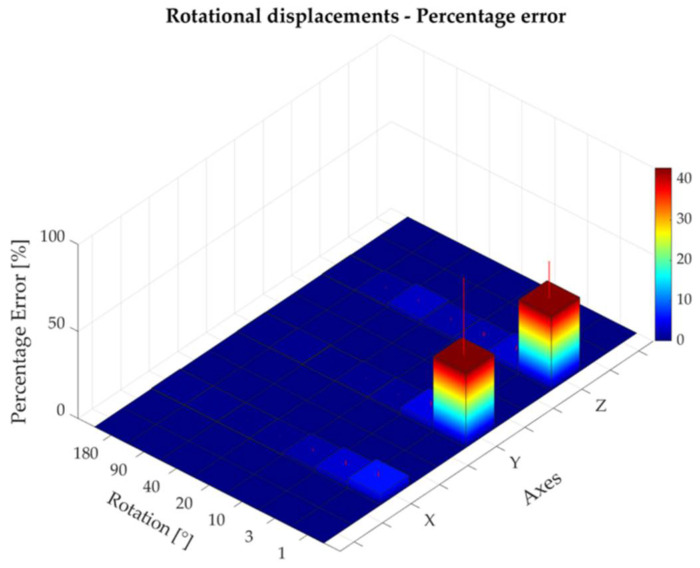
The percentage error corresponding to rotational displacements around the x-, y-, and z-axes.

**Figure 11:**
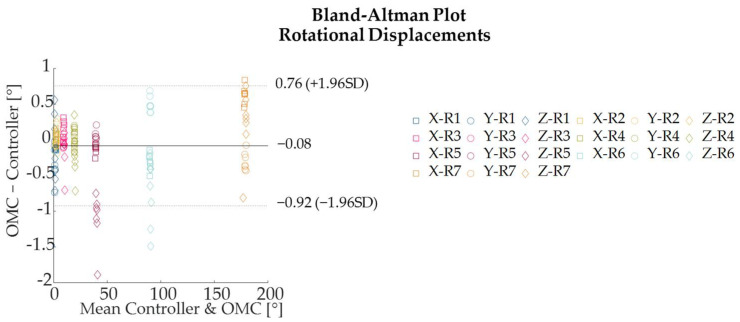
Bland–Altman plots comparing the rotational displacements measured by the Qualisys Optical Motion Capture (OMC) system and by the Controller A of the Oculus Quest 2.

The absolute and percentage errors corresponding to all the rotational displacements performed around the three axes are reported in Table 3. The maximum absolute error—expressed as mean ± standard deviation—was found to be 1.11 ± 0.37° for a rotation of 40° around the z-axis (Figure 9). The higher percentage errors were found to be 5.9%, 42.9%, and 42.6% for a rotation of 1° around the x-, y-, and z-axis, respectively (Table 3, Figure 10). The rotational displacements of 1° around all axes corresponded to a small absolute error of 0.10 ± 0.05 mm, 0.31 ± 0.25 mm, and 0.43 ± 0.19 mm for the x-, y-, and z-axes, respectively. Bland–Altman analysis showed acceptable agreement between the two measurement systems with LOAs within 1.7° and MOD equal to −0.08° (Figure 11).

## 4. Discussion

VR has been proven to be a promising technological tool for clinical applications and rehabilitation treatments, as demonstrated by the growing number of publications in cases of stroke (25.8%), brain injury (15.3%), musculoskeletal disorders (14.9%), and cerebral palsy (10.5%) [30]. Clinical interest in VR is justified by the positive impacts on patients’ motivation and engagement compared to the traditional methods for kinematics analysis [31].

Since Oculus Quest 2 is a VR tool originally designed for video games, before being used in clinical scenarios, the evaluation of its performance in terms of translational and rotational accuracy is mandatory. The performed measurements were intended to determine if Oculus Quest 2, which employs inside-out tracking, is accurate enough to measure position and orientation, as they would like to apply this VR system in the future during rehabilitation sessions of patients with shoulder musculoskeletal disorders.

Regarding the validity of our results, we approached this analysis as previous works in the literature, which assessed the rotational and translational tracking accuracy of the controllers of VR devices, defining experimental setups in which the position and orientation of the controller can be varied by known increments [23,24,32]. The common denominator of these studies is the future application of VR devices in industrial, research, or clinical settings. The authors provide a preliminary assessment of the device performance (e.g., accuracy and precision) before they can be translated into real-world application contexts. A recent study investigating the performance of Oculus Rift S, based on the same inside-out tracking of the Oculus Quest 2, showed a translational accuracy of 4.36 ± 2.91 mm and a rotational accuracy of 1.13 ± 1.23° for the controller [23]. These results are comparable with those obtained in our study, although we obtained a larger absolute error than the translational accuracy obtained in [23]. In particular, the higher absolute error of 13.52 ± 6.57 mm was registered at a distance of 500 mm from the HMD along the x-direction, corresponding approximately to the length achieved by a full elbow extension. This absolute error corresponds to a percentage error equals to 2.7%. Because the functioning of Oculus Quest 2 controllers depends on the camera sensors embedded within the HMD, such results were expected. The authors in [23] stated that the HMD’s wearer observed the controller from a close position to achieve the best performance for the controller in terms of tracking [23], contrary to our study in which the distance between HMD and controller varied. Translational increments were chosen to cover as much of the anthropometric measurements of the target population as possible, i.e., from a minimum distance corresponding to the forearm length of the 5th percentile female to a distance corresponding to the upper limb length, excluding the hand, of the 95th percentile male [26,27]. In our study, the maximum absolute error for rotational displacements was found to be 1.11 ± 0.37° for a rotation of 40° around the z-axis. During the experimental tests for rotational accuracy evaluation, we kept the controller at a distance of about 700 mm from the HMD worn by the HV, i.e., placing ourselves in the worst case (maximum distance) so as not to invalidate the results if we had considered shorter distances. In addition, the maximum error value found for the translational accuracy of the Oculus Quest 2 controller (i.e., 13.52 ± 6.57 mm) seems to be acceptable for monitoring shoulder joint angles. Indeed, considering that the user holds the controller as a hand-effector, if we consider the 95th percentile male upper arm length of 700 mm (i.e., arm fully extended), a displacement of about 20 mm of the hand-effector corresponds to an angular value of 1.64°. This value is comparable with the maximum mean absolute error for rotational accuracy obtained in our study, and it is inferior to error values found for Oculus Rift S controller and shoulder joint angles accuracy determined with a Kinect-based motion capture system [23,33]. The results of our study are acceptable for the final application of interest, namely performing rehabilitation exercises for patients with shoulder joint diseases (e.g., rotator cuff tear, frozen shoulder, scapular dyskinesis). In particular, the good accuracy obtained in both translational and rotational displacements in static conditions can be considered a solid baseline for future works in which the assessment of the VR device in dynamic conditions during upper limb movements will be performed.

Prior studies have investigated the positional and translational accuracy of other VR systems, such as the HTC VIVE and Oculus Touch controllers, which use an inside-in tracking system [24,32,34]. If compared with the results of our study, the rotational and translational accuracy of the inside-in VR systems are superior. For example, Spitzley et al. [32] reported a rotational and translational accuracy of the HTC Vive controller below 0.4° and 3 mm, respectively. Although these results are better than those obtained for the Oculus Quest 2 controller, the latter remains a viable alternative for upper limb tracking in clinical settings. Indeed, being a VR device that uses an inside-out tracking technology, Oculus Quest 2 does not require external devices for tracking the HMD and controllers, and it is a wireless VR system. For this reason, Oculus Quest 2 ensures high mobility of use without needing a limited workspace.

Obtaining quantitative information about the kinematics of patients with shoulder musculoskeletal impairments is of great interest to clinicians and patients themselves [13,14,35]. In recent years, a growing interest has been devoted to unobtrusive wearable systems for joint kinematics monitoring [36,37,38]. The quantitative evaluation and tracking of human joint movements could be relevant in different applications fields ranging from rehabilitation to sports medicine to provide data about rehabilitation progress or athlete’s performance. Thanks to the technological advancements in electronics, hardware, and computational software, the future of VR in clinical settings is spreading rapidly because of its low invasiveness and, at the same time, high user involvement. Indeed, the gamification offered by the VR-based rehabilitation systems could intensify patients’ motivation to participate in rehabilitation actively by having a clear goal to achieve during therapy [8,39]. The immersive experience offered by VR devices can provide patients with visual, vibrotactile, or auditory feedback on the performance or achievement of a given task [7]. The difficulty level of the rehabilitative exercises should be tuned based on the patients’ joint function to provide a satisfying virtual experience and, at the same time, adapted to the type of pathology being treated. Future studies on Oculus Quest 2 for VR applications in clinical settings will be devoted to evaluating motion and rehabilitation exercises in subjects with restricted movement patterns, such as those of patients following traumatic or degenerative events affecting the shoulder joint.

## 5. Conclusions

In conclusion, our study assessed the accuracy of the Oculus Quest 2 in measuring translational and rotational displacements within a range covering the whole values of interest for applications in shoulder rehabilitation. Data are promising, since the accuracy was acceptable for future measurements of upper limb movements during rehabilitative exercises performed by patients with shoulder musculoskeletal diseases. The inside-out movement tracking of Oculus Quest 2 yields an easy virtual experience, because the user’s movements are not hindered by external tracking devices during immersion in the virtual environment; in addition, the excellent cost–performance ratio makes Oculus Quest 2 a viable alternative to traditional motion analysis systems used in clinical settings to evaluate patients progress during rehabilitation.

## Figures and Tables

**Table 1:** Main features of Oculus Quest 2.

**Memory**	6 GB
**Storage**	256 GB
**Display**	LCD 1832 × 1920 per eye @ 72–120 Hz
**Graphics**	Adreno 650 (~1.2 TFLOPS)
**Sound**	2 built in speaker/3.5 mm headphone jack
**Input**	6DOF Inside-out tracking through 4 built-in cameras and 2 controllers with accelerometers and gyroscopes Optional: QWERTY keyboard (via Bluetooth)
**Controller Input**	Oculus Touch
**Camera**	4 infrared cameras
**Mass**	503 g

**Table 2:** Errors for all the translational displacements along the x-, y-, and z- directions.

Directional Axis	Translational Displacement P [mm]		Absolute Error Mean (SD) [mm]	Percentage Error Mean (SD) [%]
X	P1	5	1.38 (0.85)	29.5 (18.5)
	P2	20	2.13 (0.88)	11.0 (4.4)
	P3	70	2.22 (1.47)	3.2 (2.1)
	P4	150	4.04 (3.22)	2.7 (2.1)
	P5	250	2.41 (1.92)	1.0 (0.8)
	P6	255	2.03 (1.72)	0.8 (0.7)
	P7	270	3.21 (1.96)	1.2 (0.7)
	P8	320	5.11 (4.39)	1.6 (1.4)
	P9	400	7.06 (3.99)	1.8 (1.0)
	P10	500	13.52 (6.57)	2.7 (1.3)
	P11	505	11.67 (4.21)	2.3 (0.8)
Y	P1	5	0.55 (0.47)	11.8 (10.4)
	P2	20	1.35 (0.94)	6.0 (3.7)
	P3	70	1.68 (1.36)	2.4 (1.9)
	P4	150	2.68 (1.47)	1.8 (1.0)
	P5	250	4.22 (2.35)	1.7 (0.9)
	P6	255	4.43 (2.84)	1.7 (1.1)
	P7	270	4.07 (2.51)	1.5 (0.9)
	P8	320	5.02 (3.32)	1.6 (1.0)
	P9	400	7.11 (3.02)	1.8 (0.8)
	P10	500	8.63 (3.74)	1.7 (0.8)
	P11	505	9.74 (5.65)	1.9 (1.1)
Z	P1	5	0.81 (0.73)	16.7 (16.4)
	P2	20	1.16 (0.52)	8.5 (3.9)
	P3	70	1.16 (0.57)	1.8 (0.9)
	P4	150	1.85 (1.48)	1.3 (1.0)
	P5	250	3.59 (2.12)	1.5 (0.9)
	P6	255	3.21 (2.51)	1.3 (1.0)
	P7	270	4.17 (3.72)	1.6 (1.4)
	P8	320	4.93 (4.60)	1.6 (1.5)
	P9	400	5.50 (4.43)	1.4 (1.1)
	P10	500	7.44 (4.78)	1.5 (1.0)
	P11	505	7.60 (5.56)	1.5 (1.1)

**Table 3:** Errors for all the rotational displacements around the x-, y-, and z-axes.

Directional Axis	Rotational Displacement R [°]		Absolute Error Mean (SD) [°]	Percentage Error Mean (SD) [%]
X	R1	1	0.10 (0.05)	5.9 (3.1)
	R2	3	0.07 (0.05)	3.3 (2.4)
	R3	10	0.15 (0.12)	1.6 (1.2)
	R4	20	0.09 (0.06)	0.5 (0.3)
	R5	40	0.13 (0.07)	0.3 (0.2)
	R6	90	0.30 (0.12)	0.3 (0.1)
	R7	180	0.63 (0.13)	0.4 (0.1)
Y	R1	1	0.31 (0.25)	42.9 (44.6)
	R2	3	0.13 (0.10)	4.2 (3.0)
	R3	10	0.08 (0.04)	0.9 (0.4)
	R4	20	0.12 (0.06)	0.6 (0.3)
	R5	40	0.07 (0.07)	0.2 (0.2)
	R6	90	0.50 (0.11)	0.6 (0.1)
	R7	180	0.30 (0.13)	0.2 (0.1)
Z	R1	1	0.43 (0.19)	42.6 (21.5)
	R2	3	0.09 (0.08)	3.2 (2.6)
	R3	10	0.18 (0.24)	1.8 (2.4)
	R4	20	0.31 (0.22)	1.6 (1.1)
	R5	40	1.11 (0.37)	2.8 (0.9)
	R6	90	0.75 (0.48)	0.8 (0.5)
	R7	180	0.44 (0.27)	0.2 (0.2)

## Data Availability

The data presented in this study are available on request from the corresponding author.
